# An uncommon case of anorectal malignant melanoma (ARMM): Clinical presentation and surgical outcome

**DOI:** 10.1016/j.ijscr.2024.110394

**Published:** 2024-10-01

**Authors:** Jing Yuan Wong, Ko-Ping Tiang, Nora Binti Abdul Aziz

**Affiliations:** a Department of Surgery, University of Malaya Medical Centre, Kuala Lumpur, Malaysia; b Department of Surgery, Faculty of Medicine, Universiti Malaya, Kuala Lumpur, Malaysia

**Keywords:** Malignant melanoma, Altered bowel habit, Pelvic exenteration

## Abstract

**Introduction and importance:**

Anorectal mucosal melanoma (ARMM) is a rare disease with a poor prognosis. However, surgery is often difficult, due to the lentiginous growth pattern of such melanoma.

**Case presentation:**

A 61 years old lady presented with anal pain for 1 year, associated with painless fresh per rectal bleeding post defecation and altered bowel habit. Physical examination showed hyperpigmentation at the anal verge, extending to the dentate line. CT, MRI and PET imaging showed localised disease. She underwent pelvic exanteration and radical lymph node dissection with gracilis flap coverage. Post operatively, she recovers well, and was discharged well on day 8. HPE came back as malignant melanoma, with 1 out 12 lymph nodes involved. She was subsequently referred to oncology, started on pembrolizumab immunotherapy.

**Clinical discussion:**

Anorectal melanoma is an aggressive disease, often present with delayed diagnosis. Multiple imaging has been proposed, however none is standardized to diagnose ARMM. Immunohistochemical stains such as S-100 protein, MelanA and tyrosinase and with HMB-45 help in diagnosis and are sensitive for melanocytic differentiation. Surgery excision remains the most common and superior initial treatment for ARMM.

One retrospective study done to compare different treatment modalities has shown that patients with surgical excision and radiation therapy had the highest median survival at 32.3 months but surgical excision remains the single best modality for ARMM.

**Conclusion:**

Suspicious hyperpigmentation at the anal region should raise clinical awareness. Surgical excision with optimal margin is indicated to achieve favourable symptom control, reduce local recurrence and improve survival rate.

## Highlights

• Anorectal mucosal melanoma (ARMM) is a rare disease with a poor prognosis.
• AMM often widely metastasises at the time of diagnosis.
• Wide local excision is surgery of choice if feasible.

## 1 Introduction

Anal malignant melanoma (AMM) is a rare and aggressive malignancy, accounting for approximately 0.05 % of all colorectal malignancies and 1 % of all anal canal cancer. There are no identifiable risk factors and initial presentation of AMM could include painless PR bleed, anorectal pain, or changes in bowel habit, which are often misdiagnosed as haemorrhoids [3]. It carries a poor prognosis with a 5-year survival rate of 26.7 % in localised disease, 9.8 % in cases with regional lymph node involvement and 0 % in metastatic disease. Due to its aggressive nature, AMM often widely metastasises at the time of diagnosis. Diagnostic evaluation includes rectal examination, colonoscopy, tissue biopsy and imaging studies (PET, CT-TAP and MRI) [1,2]. It is often misdiagnosed as haemorrhoids, polyps or colorectal cancer. One of the retrospective studies showed that surgical excision is the superior single modality and could be classified into extensive resection and local resection [2]. However, several studies have shown no clear survival benefit when comparing both surgical modalities and hence, the patient's quality of life and local control of symptoms remain the principle of treatment for ARMM [4,5]. This report aims to improve the understanding and management of this rare disease. In the case of localised ARMM, the intention of surgical resection should be curative and done within 2 weeks of diagnosis, to improve the patient's quality of life by achieving better symptom control, prolong the patient's median survival age, and reduce the local recurrence rate by attaining optimal margin of resection in ARMM surgery.

## 2 Case

The patient is a 61 years old lady who presented with anal pain for 1 year, associated with painless fresh per rectal bleeding post defecation for 6 months and altered bowel habits for 2 months. Otherwise, there were no signs or symptoms of colonic obstruction, no constitutional symptoms, and no known familial history of malignancy. On examination, the patient is alert, not cachexic looking with good hydration status. Abdominal examination was normal and DRE showed a polyp at 11 o'clock of the anal verge, with patches of hyperpigmentation between 10 and 1 o'clock of the anal verge. Proctoscope reveals extension of hyperpigmentation at 10 o'clock of anal verge until anal mucosa at 11 o'clock. No mass was seen—tumour markers within normal range.

**Fig. 1:**
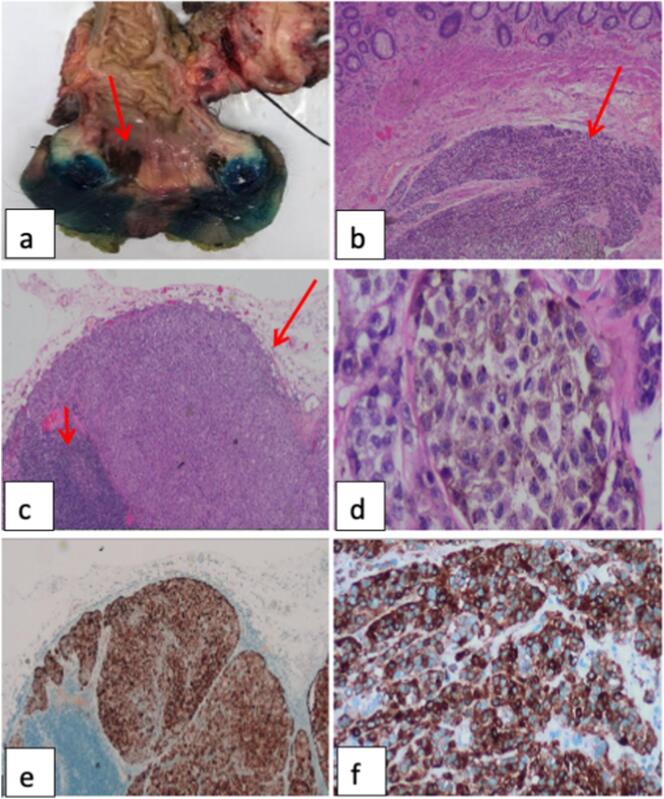
(a) Pigmented lesion (arrow). (b) Ano-rectal region pigmented lesion (arrow) H&E ×100. (c) Lymph nodes from the inguinal region show metastatic disease (long arrow), lymphoid follicle (short arrow) H&E ×200. (d) Nests of tumour cells composed of pleomorphic malignant cells exhibit vesicular to hyperchromatic nuclei set in variable amounts of cytoplasm containing an irregular distribution of pigments. Mitotic figures are frequent H&E ×400. (e) HMB45 cytoplasmic expression in metastatic clusters, lymph node ×200. (f) Malignant melanocytes staining for HMB45 immunohistochemistry in the ano-rectal tissue ×400.

She was scheduled for examination under anaesthesia which showed a pedunculated polyp with a short stalk measuring 1 cm at the 11 o'clock position of the anal verge, hyperpigmented skin lesion from 3 to 9 o'clock position of the anal verge, extending to the dentate line, with hyperpigmented skin lesion on the haemorrhoidal plexus at 12 o'clock. Anal polyp and hyperpigmented haemorrhoidal plexus were excised and sent for HPE, revealing anal malignant melanoma. CT, MRI and PET imaging were done and showed localised disease. She underwent posterior Pelvic Exenteration, open abdominoperineal resection (APR), Bilateral Inguinal Lymph Node Dissection, and wound Coverage with bilateral gracilis flap. HPE showed a dark pigmented area was seen at the recto-anal junction involving the superficial rectal mucosa and anal skin. There was no protruding tumour nodule or ulceration. The surface mucosa was intact (Fig. 1a).

Haematoxylin and eosin stains of the formalin-fixed paraffin-embedded specimen confirmed anorectal lesional tissue composed of nests of pigmented malignant cells (Fig. 1b) with metastatic disease seen in regional lymph node (Fig. 1c). Nests of neoplastic cells are also seen within the dermis of the anus. Neoplastic clusters are composed of malignant cells that exhibit mild to moderate nuclear pleomorphism, and occasional prominent nucleoli with finely granular cytoplasmic melanin. Mitotic figures are frequently encountered (Fig. 1d). On immunohistochemistry, tumour cells were diffusely positive for HMB45 (Fig. 1e, f). These findings were consistent with a malignant melanocytic tumour.

Post operatively, she recovers well, able to ambulate postoperative day 5, and was discharged well on day 8. During routine follow up in the clinic, HPE came back as malignant melanoma, with 1 out 12 lymph nodes involved. She was subsequently referred to oncology, which was then started with pembrolizumab immunotherapy. This work has been reported in line with the SCARE criteria [20].

## 3 Discussion

Anorectal melanoma is an uncommon and aggressive mucosal type of melanocytic malignancy. It is often misdiagnosed as haemorrhoids, or polyps and rectal cancer [8]. Embryologically, melanocytes arise from neuro-ectodermal multipotent neural crest cells, and can be found in anal squamous zone, sometimes in the anal transitional zone [9]. It is often difficult to diagnose due to the hidden site. According to a study in European journal of cancer, 444 patients with mucosal melanoma were followed up, anorectal malignant melanoma is aggressive in nature, and patients often present with poor prognosis, which is related to delay in diagnosis [10].

Multiple imaging has been proposed, however none is standardized to diagnose ARMM. In our patient, proctosigmoidoscopy allows macroscopic identification of polyp mass, and brown pigment stain in which tissue diagnosis can be obtained. Endoscopic ultrasound and MRI, have been advocated to supplement colonoscopy, to allow evaluation of tumour infiltration, and assessment of local regional lymph nodes. PET/Contrast-enhanced CT have been mainly studied for the evaluation of loco-regional and systemic involvement, and found to be superior in terms of staging. At present, there is no specific staging classification for anorectal melanoma which provides prognostic value [11,13].

Microscopically, pigmented lesions of anorectal tract are highly suspicious of melanoma. Chute described in her study there were 4 histology cell types observed: epithelioid, spindle cell, lymphoma-like and pleomorphic. Majority of cases showed >1 cell types, and less than half showed significant pigmentation, and more than half were amelanotic. Immunohistochemical stains such as S-100 protein, MelanA and tyrosinase and with HMB-45 help in diagnosis and sensitive for melanocytic differentiation [12].

Surgery excision remains the most common and superior initial treatment for ARMM.

One retrospective study done to compare different treatment modalities has shown that patients with surgical excision and radiation therapy had the highest median survival at 32.3 months but surgical excision remains the single best modality for ARMM. Immediate surgical intervention should be offered within 2 weeks of discovering suspicious pigmented lesions as it may improve the overall survival rate. The 2 most common surgeries for ARMM are abdominoperineal resection (APR) and wide local excision (WLE), aiming to achieve optimal resection margin. There had been a long-standing debate between choices of surgery: WLE vs APR as several studies showed that both of the surgeries didn't improve the survival median age of patients [14].

However, in one of the studies, the general resection margins recommended are 1 mm tumour = 1 cm margin; and 1 to 4 mm tumour = 2 cm margin. If the tumour is >4 mm, an APR is recommended [3]. Despite the controversy, a study in one centre showed that APR is superior as 62.5 % of patients who underwent WLE in this study developed local recurrence requiring further intervention [6]. Furthermore, APR may provide substantial benefits by avoiding severe symptoms such as incontinence and continuous bleeding of the tumour, which may improve a patient's quality of life post-surgery [7].

In order to achieve R0, lymph node clearance has been a subject of debate. Goldman et al. showed that nodal involvement in AM did not predict outcome of patients undergoing radical resection. Bilateral inguinal lymphadenopathy in clinically not palpable lymph nodes did not improve survival, but increased risk of complications such as lymphoedema [[14], [15], [16]].

Due to ARMM's low incidence, there have been no randomized controlled trials to evaluate the most effective means of control, timing for surgery, and proper guidelines in determining the extent of resection in different stages of the disease. In a recent meta-analysis done, surgical approaches and neoadjuvant therapies to ARMM are evolving and current data are still lacking on whether resection margins were microscopically negative (R0) and rate of local recurrence, which might influence the median survival age.

With the introduction of targeted therapy, more therapeutic choices are available for ARMM patients. 40–50 % of melanoma shows BRAF-V600, therapies such BRAF plus MEK inhibitors shown to improve patient outcomes [[17], [18], [19]]. Immunotherapy has demonstrated the ability to reduce the risk of recurrence for melanoma following surgery resection, and also improve survival in patients with advanced malignancy. The development of immune checkpoint inhibitors (ICI), oncolytic virus therapy, and modulators of tumour microenvironemnt are crucial in improving quality of life for patients. Monoclonal antibodies PD-1 inhibitors such as Pembrolizumab (Keytruda) and nivolumb (Opdivo) can be used for advanced melanoma, or as adjuvant treatment.

## 4 Conclusion

ARMM is a rare subtype of melanoma with aggressive behaviour and poor prognosis, which requires high clinical suspicion of ARMM. Surgical excision with optimal margins of resection is recommended as treatment for either curative intent or palliation symptomatic control. New therapies such as immunotherapy, which help to improve the prognosis of such rare disease especially in post surgery intervention or patients in advanced stage.

## Abbreviations

ARMM: anorectal mucosal melanoma
PR bleed: per rectum bleed
DRE: digital rectal examination
WLE: wide local excision
APR: abdominoperineal excision

## Consent

Written consent was obtained from the patient and all participants for the publication of this case report and accompanying images. A copy of the written consent is available for review by the Editor-in-Chief of this journal on request.

## Ethical approval

Ethics approval was not required from the institution for the publication of this case report. We have adhered to ethical standards by ensuring patient confidentiality and obtaining informed consent, and waiving of consent by UMMC-Medical Research Ethics Committee.

## Funding

This research did not receive any specific grant from funding agencies in the public, commercial, or not-for-profit sectors.

## Author contribution

JY Wong and KP Tiang were responsible for the write-up.

Aziz Nora was responsible for overseeing the write-up as a supervisor.

## Guarantor

Ko-Ping Tiang will be the guarantor.

## Conflict of interest statement

There are no conflicts of interest to declare.
